# Location of Receipt of Initial Treatment and Outcomes in Long-Term Breast Cancer Survivors

**DOI:** 10.1371/journal.pone.0170081

**Published:** 2017-01-13

**Authors:** Arup K. Sinha, Jenil R. Patel, Yu Shen, Naoto T. Ueno, Sharon H. Giordano, Debu Tripathy, David S. Lopez, Carlos H. Barcenas

**Affiliations:** 1 Department of Breast Medical Oncology, The University of Texas MD Anderson Cancer Center, Houston, Texas, United States of America; 2 Department of Biostatistics, The University of Texas School of Public Health, Houston, Texas, United States of America; 3 Division of Epidemiology, Human Genetics and Environmental Sciences, The University of Texas School of Public Health, Houston, Texas, United States of America; 4 Department of Biostatistics, The University of Texas MD Anderson Cancer Center, Houston, Texas, United States of America; 5 Department of Health Services Research, The University of Texas MD Anderson Cancer Center, Houston, Texas, United States of America; West Virginia University, UNITED STATES

## Abstract

**Purpose:**

Cancer outcomes differ depending on where treatment is received. We assessed differences in outcomes in long-term breast cancer survivors at a specialty care hospital by location of their initial treatment.

**Methods:**

We retrospectively examined a cohort of women diagnosed with invasive early-stage breast cancer who did not experience recurrence for at least 5 years after the date of diagnosis and were evaluated at The University of Texas MD Anderson Cancer Center between January 1997 and August 2008. The location of initial treatment was categorized as MD Anderson (MDA-treated) or other (OTH-treated). Outcomes analyzed included recurrence-free survival (RFS), distant relapse-free survival (DRFS), and overall survival (OS). The Kaplan-Meier product-limit method was used to compare outcomes between the two groups. Cox proportional hazards models were used to estimate hazard ratios (HR) and 95% confidence intervals (CI).

**Results:**

We identified 5,091 breast cancer survivors (median follow-up 8.6 years), of whom 89.1% were MDA-treated. The 10-year OS, RFS, and DRFS rates were 90.9%, 88.4%, and 89.0% in the MDA-treated group and 74.3%, 49.8%, and 52.7% in the OTH-treated group, respectively. We observed worse outcomes in the OTH-group in both the univariate analysis and the multivariable analysis (OS: HR = 4.8, 95% CI = 3.9–6.0; RFS: HR = 5.8, 95% CI = 4.8–7.0; DRFS: HR = 5.4, 95% CI = 4.5–6.6).

**Conclusion:**

Long-term breast cancer survivors who initiated their treatment at MD Anderson had better outcomes. Location of initial treatment could be an independent risk factor for survival outcomes at specialty care hospitals. This analysis has limitations inherent to retrospective observational studies such as other unmeasured variables may be associated with worse prognosis.

## Introduction

The impact of the location or hospital setting where a cancer patient receives treatment on the patient’s health outcomes has been studied with great interest owing to concerns about cost and quality of care [1–3]. Multiple studies have shown that high-volume hospitals, high physician volume, and specialization are associated with better cancer outcomes [4,5]. A recent study reported that, across all cancer types, the 1-year mortality rate in specialty care hospitals was 10% lower than in community hospitals, and this pattern remained consistent for up to 5 years [6]. However, specialty care hospitals commonly treat a heterogeneous group of patients where some patients may receive their initial treatment within these specialty care hospitals and others may initiate their treatment in community hospitals and eventually establish follow-up care in the specialty care hospital. This heterogeneity of location of initial treatment could potentially lead to substantial differences in outcomes within a specialty care hospital.

Breast cancer (BC) survivors are still at risk of disease recurrence and death 5 years after diagnosis [7]. Therefore, in the current study, we sought to determine whether the location of receipt of the initial treatment affected late (>5 years) cancer outcomes among BC survivors who had presented to a single specialty care hospital and had not experienced recurrence for at least 5 years after diagnosis. Specifically, we assessed recurrence-free survival (RFS), distant relapse-free survival (DRFS), and overall survival (OS).

## Methods

### Study population and hypothesis

This retrospective cohort consisted of women diagnosed with invasive early-stage (I-III) BC who had presented to The University of Texas MD Anderson Cancer Center between January 1997 and August 2008 and had not experienced any recurrence for at least 5 years after the date of diagnosis. This cohort was identified from the Breast Cancer Management System database housed in the Department of Breast Medical Oncology at MD Anderson, which contains records for all BC patients who have presented to MD Anderson since January 1, 1997. For the purpose of this study, the BC survivors were categorized into one of two groups, based on the location where initial treatment was received: 1) those who had received their initial treatment—chemotherapy, surgery, or radiation—at MD Anderson (MDA-treated) or 2) those who had received their initial treatment at a facility other than MD Anderson (OTH-treated) but had presented to MD Anderson at some point in their care for any reason, including but not limited to second opinions or transfer of care to MD Anderson. We hypothesized that survival outcomes would differ between these two groups of BC survivors. Keeping in mind that the inclusion of survivors presenting to MD Anderson 5 years or more after the initial BC diagnosis could introduce bias by design if a substantial number of them presented with disease recurrence, we also conducted a separate analysis excluding such survivors.

### Measurements of exposure, confounders, and outcomes

A BC survivor was considered MDA-treated if 1) she had been diagnosed with invasive BC within 180 days before her initial consultation visit to MD Anderson with one of the BC subspecialists (in medical oncology, radiation oncology, or surgical oncology), and 2) she had received initial treatment for BC at MD Anderson. This specific categorization has been used previously by others [8]. BC survivors who did not meet at least one of these criteria were categorized as OTH-treated. Standardized Definitions for Efficacy Endpoints in Adjuvant Breast Cancer Trials criteria were used to define the outcomes of interest [9]. RFS was measured from the date of diagnosis of the primary cancer to the date of the first invasive ipsilateral breast tumor recurrence, local or regional invasive recurrence, distant recurrence, or death from any cause. DRFS was measured from the date of diagnosis of primary cancer to the date of first distant recurrence or death from any cause. Patients were censored at the last day of follow-up.

We collected demographic factors such as age at diagnosis, year of diagnosis, race/ ethnicity (self-reported), and menopausal status at diagnosis. We classified the tumors as hormone receptor-positive if the tumor was estrogen receptor (ER)- or progesterone receptor (PR)-positive and hormone receptor-negative if the tumor was both ER- and PR-negative, as determined by immunohistochemistry staining using institutional laboratory thresholds. Human epidermal growth factor receptor 2 (HER2) status was determined by immunohistochemistry or fluorescence in situ hybridization. We grouped the tumors into one of three categories: 1) hormone receptor-positive (ER- or PR-positive and HER2-negative or unknown), 2) triple-negative (ER- and PR-negative and HER2-negative or unknown), or 3) HER2-positive (independent of ER or PR status). Tumor stage was determined using the American Joint Committee on Cancer (AJCC) 5th edition staging method for patients diagnosed before 2003 and the AJCC 6th edition for patients diagnosed in 2003 or later. Tumor grade and histologic findings were extracted from biopsy and surgical pathology reports. We captured treatments received, such as chemotherapy, surgery, radiation therapy, and endocrine therapy, by reviewing the patient’s medical record. We determined the most recent vital status of each patient using data from the Tumor Registry at MD Anderson and from medical records. We received institutional review board approval to conduct this study (PA13-0424).

### Statistical analyses

Demographic characteristics were summarized and compared using chi-square analysis or the *t* test, as appropriate. We used the Kaplan-Meier product-limit method to estimate the survival functions. The survival function provides the probability of observing a survival time greater than or equal to a specified time. Without imposing any parametric assumption, Kaplan-Meier estimator of survival function is obtained by multiplying a sequence of conditional survival probabilities with information from both uncensored and censored observations. We used log-rank statistic to test the equality of survival functions by group. The log-rank test compares the observed and expected number of events across all failure time points. Cox proportional hazards regression was used to estimate the effect sizes of covariates on the hazard of failure, where the hazard function measures the instantaneous rate of an event to occur, given the individual has survived till that time point. We used univariate Cox proportional hazards regression models to evaluate the crude associations between the main exposure of interest (i.e., MDA-treated or OTH-treated) and potential confounding variables and the outcomes of interest. Variables that showed significant association in the univariate log-rank test were considered potential confounders and were entered in the multivariable Cox proportional hazards model. A backward selection process was used and significant variables were retained in the final model. We used a complete case analysis to handle missing values. All statistical tests were performed with a two-sided significance level of 0.05. Statistical analyses were performed using SAS software, version 9.4 (SAS Institute, Cary, North Carolina), and STATA software, version 12 (Statacorp, College Station, Texas).

## Results

We identified 5,091 female long-term BC survivors with a median follow-up period of 8.6 years, of whom 4,534 (89.1%) were MDA-treated (median follow-up period 8.6 years) and 557 (10.9%) were OTH-treated (median follow-up period 8.9 years). In the OTH-treated group, 131 (23.5%) did not receive their initial treatment for BC at MD Anderson despite having a consultation visit at the institution within 180 days after diagnosis, likely representing those who presented for a second opinion; 195 (35.0%) had a consultation visit at MD Anderson at 180 or more days after diagnosis, but did receive some treatment at MD Anderson; and 231 (41.5%) neither received their initial treatment at MD Anderson nor visited MD Anderson within 180 days after diagnosis.

The demographic and clinical characteristics at diagnosis for the overall cohort and by group are presented in Table 1. Briefly, the mean age at diagnosis for the total cohort was 53.3 years, and most were white (73.2%), around half were diagnosed with stage I disease (52.4%), most had hormone receptor-positive tumors (72.5%), and most had received chemotherapy (66.2%), endocrine therapy (72.9%), or radiation therapy (66.7%). Compared with the MDA-treated group, those in the OTH-treated group were younger at diagnosis, and less likely to be white, and less likely to be postmenopausal. They had more advanced disease, had more triple-negative tumors, were less likely to have received endocrine therapy, were more likely to have received chemotherapy, and were more likely to have received radiation therapy compared with the MDA-treated group.

**Table 1:** Demographic and clinical characteristics of breast cancer survivors who received their initial treatment at our institution (MDA-treated) or elsewhere (OTH-treated).

Characteristic	No. (%)			p value^a^
	Total cohort, n = 5091	MDA-treated, n = 4534	OTH-treated, n = 557	
Age at diagnosis				0.044
35 years or younger	270 (5.3)	238 (5.2)	32 (5.8)	
36–59 years	3357 (65.9)	2967 (65.4)	390 (70.0)	
60 years or older	1464 (28.8)	1329 (29.3)	135 (24.2)	
Mean age at diagnosis (standard deviation)	53.3 years (11.7 years)	53.6 years (11.6 years)	51.1 years (11.7 years)	<0.001
Year of diagnosis				<0.001
1995 or earlier	75 (1.5)	0 (0.0)	75 (13.5)	
1996–2000	1508 (29.6)	1312 (28.9)	196 (35.2)	
2001–2005	2541 (50.0)	2265 (50.0)	276 (50.0)	
2006 or later	967 (19.0)	957 (21.1)	10 (1.8)	
Race/ethnicity				<0.001
White	3727 (73.2)	3356 (74.0)	371 (66.6)	
Black	506 (9.9)	421 (9.3)	85 (15.3)	
Spanish/Hispanic	647 (12.7)	563 (12.4)	84 (15.1)	
Other	211 (4.1)	194 (4.3)	17 (3.1)	
Tumor receptor status				<0.001
Hormone receptor-positive	3691 (72.5)	3300 (72.8)	391 (70.2)	
Triple-negative	722 (14.2)	614 (13.5)	108 (19.4)	
HER2-positive	678 (13.3)	620 (13.7)	58 (10.4)	
Initial tumor stage				<0.001
0	60 (1.2)	57 (1.3)	3 (0.5)	
I	2669 (52.4)	2422 (53.4)	247 (44.3)	
II	1873 (36.8)	1660 (36.6)	213 (38.2)	
III	489 (9.6)	395 (8.7)	94 (16.9)	
Tumor grade				0.002
I	402 (7.9)	359 (7.9)	43 (7.7)	
II	2426 (47.7)	2198 (48.5)	228 (40.9)	
III	2263 (44.5)	1977 (43.6)	286 (51.3)	
Histologic findings				<0.001
Ductal	4066 (79.9)	3586 (79.1)	480 (86.2)	
Lobular	423 (8.3)	390 (8.6)	33 (5.9)	
Mixed	315 (6.2)	304 (6.7)	11 (2.0)	
Other	287 (5.6)	254 (5.6)	33 (5.9)	
Received chemotherapy	3372 (66.2)	2974 (65.6)	398 (71.5)	0.006
Received endocrine therapy	3710 (72.9)	3358 (74.1)	352 (63.2)	<0.001
Surgery				0.012
Lumpectomy	2585 (50.8)	2330 (51.4)	255 (45.8)	
Mastectomy	2506 (49.2)	2204 (48.6)	302 (54.2)	
Received radiation therapy	3395 (66.7)	2990 (65.9)	405 (72.7)	0.001

^a^ A two-sample *t* test was used to compare mean age between the groups; Pearson’s chi-square tests were used for all other comparisons.

We observed a statistically significant difference in vital status between the two groups; 29.3% of the OTH-treated group (median follow-up period 8.6 years), compared with only 7.6% of the MDA-treated group (median follow-up period 9.0 years), had died at the time of our analysis (p < 0.01). We also observed a higher rate of death as the first event in the OTH-treated group (10.2%) than in the MDA-treated group (5.3%). Distant disease recurrences were more common than local recurrences (5.8% compared with 1.6%) in the overall cohort, as well as within each group. Specific survival outcomes and recurrence events are detailed in Table 2.

**Table 2:** Survival and recurrence outcomes in breast cancer survivors who received their initial treatment at our institution (MDA-treated) or elsewhere (OTH-treated).

Outcome	No. (%)		
	Total cohort, n = 5091	MDA-treated, n = 4534	OTH-treated, n = 557
Overall survival			
Alive	4583	4189 (92.4)	394 (70.7)
Dead	508	345 (7.6)	163 (29.3)
Follow-up period			
Median (range)	8.7 years (5.0–22.3 years)	8.6 years (5.0–16.7 years)	9.0 years (5.0–22.3 years)
Interquartile range	6.6–11.2 years	6.6–11.1 years	6.8–12.8 years
Time to event^a^			
Median (range)	8.8 years (5.1–19.7 years)	8.7 years (5.1–17.0 years)	8.8 years (5.1–19.7 years)
Interquartile range	7.0–11.0 years	7.0–10.5 years	7.1–12.0 years
Recurrence-free survival			
Local recurrence	49 (1.0)	26 (0.6)	23 (4.1)
Local recurrence → death	4 (0.0)	1 (0.0)	3 (0.5)
Distant recurrence	109 (2.1)	63 (1.4)	46 (8.3)
Distant recurrence → death	187 (3.7)	98 (2.2)	89 (16.0)
Local recurrence → distant recurrence	10 (0.2)	2 (0.0)	8 (1.4)
Local recurrence → distant recurrence → death	19 (0.4)	5 (0.1)	14 (2.5)
Death only	298 (5.9)	241 (5.3)	57 (10.2)
Follow-up period			
Median (range)	8.6 years (5.0–20.1 years)	8.6 years (5.0–16.7 years)	8.9 years (5.0–20.1 years)
Interquartile range	6.6–11.1 years	6.6–11.0 years	6.8–11.6
Time to event			
Median (range)	7.5 years (5.0–20.1 years)	7.7 years (5.1–16.0 years)	7.2 years (5.0–20.1 years)
Interquartile range	6.0–7.5 years	6.2–9.7 years	5.6–9.0 years

^a^ Median time to event represents the median time to develop the first event among patients who had an event.

**Fig 1:**
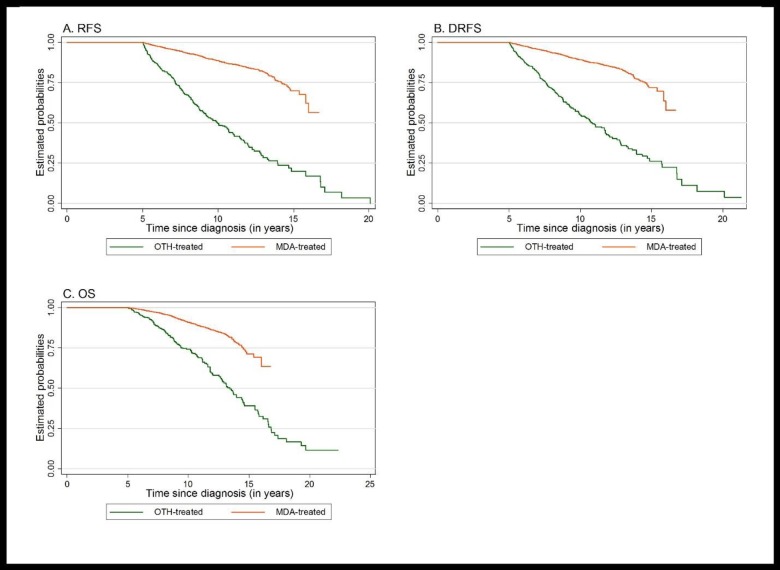
Kaplan-Meier curves for (A) recurrence-free survival (RFS), (B) distant relapse-free survival (DRFS), and (C) overall survival (OS) for breast cancer survivors who received their initial treatment at our institution (MDA-treated) or elsewhere (OTH-treated).

Fig 1 shows the Kaplan-Meier product-limit estimates of the cumulative probabilities of OS, RFS, and DRFS for both groups. The log-rank tests showed statistically significant differences in all outcomes between the groups (p < 0.01). The 10-year OS, RFS, and DRFS rates were 90.9%, 88.4%, and 89.0% in the MDA-treated group and 74.3%, 49.8%, and 52.7% in the OTH-treated group, respectively. These results indicate that the MDA-treated population had a higher chance of survival and freedom from recurrences than did the OTH-treated population.

Univariate Cox proportional hazards regression analysis showed a significantly increased risk of death (OS: hazard ratio (HR) = 3.3, 95% confidence interval (CI) = 2.7–4.0) and recurrence (RFS: HR = 5.6, 95% CI = 4.8–6.6; DRFS: HR = 5.0, 95% CI = 4.2–5.9) in the OTH-treated group compared with the MDA-treated group. When adjusted for selected confounding variables in the multivariable Cox proportional hazards model (age at diagnosis, year of diagnosis, race, stage, surgery type, chemotherapy, and endocrine therapy, as well as grade for the recurrence analyses), these risks remained for the OTH-treated population (OS: HR = 4.8, 95% CI = 3.9–6.0; RFS: HR = 5.8, 95% CI = 4.8–7.0; DRFS: HR = 5.4, 95% CI = 4.5–6.6). In the sensitivity analysis, in which patients who presented at MD Anderson 5 years or more after their initial BC diagnosis were excluded, the differences in outcomes between the groups remained statistically significant in the multivariable analysis (OS: HR = 3.6, 95% CI = 2.8–4.8; RFS: HR = 3.1, 95% CI = 2.4–4.0; DRFS: HR = 3.0, 95% CI = 2.3–3.9).

## Discussion

In this cohort of long-term (>5 years) BC survivors, we observed significant differences in demographic, clinical, pathologic, and treatment characteristics, as well as in RFS, DRFS, and OS outcomes, between the MDA-treated group and the OTH-treated group. These findings suggest that within a specialty care hospital, those initially treated outside of the specialty care hospital may have different characteristics and are likely to have poorer outcomes than those initially treated within the hospital, suggesting that location of initial treatment could be an independent risk factor for survival and recurrence.

BC survivors in the OTH-treated group had worse baseline disease prognostic factors and poorer event-free survival outcomes. Age at diagnosis, race/ethnicity, tumor stage, and receptor status are well-established prognostic factors in BC [10,11] and we found that the OTH-treated group of BC survivors had more of these risk factors than the MDA-treated group. Interestingly, although both groups remained disease-free until at least 5 years from their date of diagnosis, the OTH-treated cohort had several less favorable prognostic markers at the time of diagnosis. The 5-year benchmark in BC survivorship is crucial, because at this point in time, the care of long-term survivors is usually transferred to survivorship clinics or to a primary care physician.

The OTH-treated group was heterogeneous, consisting of some survivors who presented to MD Anderson at least 180 days after their initial BC diagnosis and others who presented to MD Anderson within 180 days but did not receive their initial treatment at MD Anderson. In fact, around 50% of the OTH-treated group presented to MD Anderson more than 1 year, 38% presented more than 2 years, and 31% presented more than 5 years after their initial BC diagnosis. Some BC survivors from this group could have presented to MD Anderson for a second opinion, and others may have relocated to Houston and eventually became part of the Breast Cancer Survivorship Clinic for their follow-up visits although they started and completed their treatment somewhere else.

The consistently worse outcomes in the OTH-treated group could potentially arise from several factors, such as transferring care to MD Anderson because of poor disease prognosis. For instance, the proportion of stage III disease was higher in the OTH-treated group than in the MDA-treated group (16.9% compared with 8.7%). We speculate that some of the survivors in the OTH-treated group who presented to MD Anderson more than 180 days after diagnosis could have had a delay in the initiation of their treatment. However, we do not have enough information to confirm this. Treatment delay is known to be associated with poor survival outcomes in BC [12]. Also, delay in treatment for BC is more common in patients with low socioeconomic status [13], which could also have influenced the outcomes in the OTH-group. It has been reported that patients of low socioeconomic status are more likely to receive treatments in low volume, non-teaching hospitals, and they are also likely to have less access to health care benefits and suffer worse outcomes [14,15]. We did not have information on socioeconomic status for our cohort and it is unknown if the OTH-treated group may have more patients with lower socioeconomic status. Given that race has been associated with socioeconomic status [16], the higher proportion of non-white survivors in the OTH-treated group could be an indication of this.

We were interested in the proportion of survivors who had presented to MD Anderson 5 years after their initial BC diagnosis because this group of individuals either presented to MD Anderson to establish follow-up care or sought treatment at MD Anderson owing to late (>5 years) disease recurrence. However, inclusion of survivors in the OTH-treated group who presented to MD Anderson with disease recurrence would bias the estimates of event-free survivals. Hence, we performed a sensitivity analysis after removing individuals who presented to MD Anderson more than 5 years after diagnosis, and this analysis still showed significantly worse outcomes in the OTH-treated group compared with the MDA-treated group. We noticed that patients in the OTH-treated group had a longer follow-up period, but there were only four patients in the OTH-treated group who had longer follow-up periods than the maximum follow-up period of the MDA-treated group. The differences in the outcomes between the groups were likely not altered by these few observations.

Heterogeneity in patient characteristics and outcomes within large single-center academic hospitals owing to referred patients has been reported before. Kokmen et al [17] compared the sociodemographic and clinical characteristics of patients with Alzheimer disease at Mayo Clinic using resources of the Rochester Epidemiology Project. The authors grouped the patients into three categories: residents of Rochester, Minnesota; patients referred to Mayo Clinic from the remainder of Minnesota and the four surrounding states; and patients referred from the remainder of the United States. They found significant differences in important patient characteristics between the groups. The authors noted that such differences in patients within an institution can limit the extrapolation of results obtained from epidemiologic studies using data from multiple institutions.

In another investigation, Riggs et al [18] investigated the reasons for substantially high in-hospital crude mortality rates due to acute stroke in a rural academic medical center. They found that the increased in-hospital mortality rate was mostly attributable to the hospital transfer patients, i.e., patients who were admitted or evaluated in another acute care setting and transferred to the hospital of interest. In a study of multiple sclerosis from a university-based referral center, significant differences were observed in epidemiologic and clinical characteristics of referred patients, who tended to be younger and to more often report worsening of disease than those in a population-based group, indicating that a university setting may not be an appropriate one for collecting generalizable natural history data for that disease [19]. Our study findings are consistent with those of these two previous studies in that patients who transferred their care to MD Anderson fared worse than patients who initiated care at MD Anderson.

The current study has some limitations, including those inherent to retrospective observational studies. For example, other unmeasured factors could be associated with worse prognosis in the OTH-treated group. In addition, the composition of patients who present to MD Anderson for treatment may not be the same as that of the other specialty care hospitals; therefore, the results of this study may not be generalizable to other specialty care hospitals. In our study, there was an imbalance in the sample sizes between the two groups. However, the findings remained consistent across all analyses despite this imbalance.

In summary, our results indicate that the survival outcomes of MDA-treated long-term BC survivors are better than those of OTH-treated patients. These differences in outcomes among patients within a single specialty care hospital are likely due to multiple factors, and further investigation is needed to establish the underlying causes.

## Supporting Information

S1 Data Location of initial treatment dataset. (XLS)

S1 File Data dictionary. (DOCX)
