# Supplementary material for: Location of Receipt of Initial Treatment and Outcomes in Long-Term Breast Cancer Survivors
Source: PLoS One. 2017 Jan 13;12(1):e0170081. doi: 10.1371/journal.pone.0170081 (PMC5234813; doi:10.1371/journal.pone.0170081)
Supplement: S1 File — (DOCX) [file pone.0170081.s002.docx]

**Accompanying data provided by: Carlos H Barcenas, MD, MS, Department of Breast Medical Oncology, MD Anderson Cancer Center, Houston, Texas.**

**General explanation of the study:**

This data is collected from the Breast Cancer Management System (BCMS) data base in the Department Brest Medical Oncology at MD Anderson Cancer Center, Houston, Texas. We are providing the only those variables used for the purpose of the manuscript “Location of receipt of initial treatment and outcomes in long-term breast cancer survivors”. This data is de-identified and do not have any patient information which are confidential. The list of variables names and variable levels are provided below.

| **Variable name** | **Variable level** |
| --- | --- |
| ID | Subject ID |
| age_at_dx | Age at diagnosis of the primary breast cancer |
| diag_year | Year of diagnosis of the primary breast cancer |
| race | Race/Ethnicity |
| hormstat | Hormone receptor status |
| stage | Stage of the primary rumor |
| grade | Grade of the primary tumor |
| histology | Histology of the primary tumor |
| chemoth | Chemotherapy received |
| hormth | Hormone therapy received |
| surgery | Type of surgery |
| radiation | Radiation received |
| time_os | Time from diagnosis of primary cancer to death or last follow-up, in years |
| c_os | Death status |
| time_rfs | Time from diagnosis of primary cancer to the time of first event of any RFS outcomes or last follow-up, in years |
| c_rfs | Recurrence free survival status |
| time_drfs | Time from diagnosis of primary cancer to the time of first event of any DRFS outcomes or last follow-up, in years |
| c_drfs | Distant recurrence free survival status |
| mda_treated | Patients MDA-treated or not |
| dx_mdavisit | Time from diagnosis of the primary breast cancer to MD Anderson visit in years |
| event_type | Type of events |
